# Cutaneous Manifestations in COVID-19–Positive African American Patients

**DOI:** 10.31486/toj.21.0057

**Published:** 2022

**Authors:** Avery Bryan, Hrishikesh Samant, Ameya Asarkar, Cherie-Ann O. Nathan, Alok Khandelwal

**Affiliations:** ^1^ Department of Otolaryngology, Head and Neck Surgery, Ochsner–Louisiana State University Health, Shreveport, LA; ^2^ Multi-Organ Transplant Institute, Ochsner Clinic Foundation, New Orleans, LA

**Keywords:** *COVID-19*, *skin abnormalities*, *skin manifestations*

## Abstract

**Background:** The United States of America is the leading country in confirmed cases of and deaths from severe acute respiratory syndrome coronavirus 2, the virus that causes coronavirus disease 2019 (COVID-19). In April and May 2020, respectively, 0.2% of patients in a Chinese COVID-19–positive cohort and 20.4% of patients in an Italian COVID-19–positive cohort developed cutaneous abnormalities. Cutaneous abnormalities associated with COVID-19 are not well documented or discussed, and investigation of cutaneous manifestations is necessary to determine if they have any clinical value.

**Methods:** We conducted a retrospective study of COVID-19–positive patients who were admitted to Ochsner–Louisiana State University-Shreveport and Ochsner–Louisiana State University-Monroe facilities in Louisiana. Cutaneous manifestations were determined from clinical notes, descriptions in medical records, and a billing code for skin rashes.

**Results:** Of 1,086 COVID-19–positive patients investigated, 871 were African American and 130 were Caucasian. Only 10 patents exhibited probable COVID-19–induced cutaneous abnormalities: 6 (0.7%) of the 871 African American patients and 4 (3.1%) of the 130 Caucasian patients. Dermatologic abnormalities included pruritic or erythematous rash and hypopigmentation of the face, upper chest, abdomen, and trunk areas. Our data are consistent with the smaller percentage of patients in the Chinese cohort study vs the larger percentage in the Italian cohort study.

**Conclusion:** Our data provide evidence that cutaneous manifestations of COVID-19, especially in African American patients, are rare, but documentation of more cases is necessary to establish a cause and effect for COVID-19–induced skin manifestations.

## INTRODUCTION

Cutaneous manifestations in coronavirus disease 2019 (COVID-19)–infected patients have been reported from around the world. In April and May 2020, respectively, 0.2% (2 of 1,099 patients) of a Chinese cohort and 20.4% (18 of 88 patients) of an Italian cohort developed cutaneous abnormalities.^1,2^ Case reports have been published highlighting cutaneous manifestations in COVID-19–positive patients,^3-7^ but to our knowledge, no comprehensive COVID-19–induced cutaneous manifestation studies have been reported from the United States. Further, the patient populations in the majority of published studies have Fitzpatrick type 1, 2, and 3 integument, necessitating the need to document COVID-19 skin manifestations in African Americans. Documenting cutaneous abnormalities present at diagnosis and during the course of COVID-19 in as many patients as possible may reveal if these cutaneous manifestations have any clinical value and may provide clinical clues to help guide diagnosis and treatment.

## METHODS

The Louisiana State University Health-Shreveport Institutional Review Board approved this study. We conducted a retrospective medical records review of patients admitted to Ochsner–Louisiana State University-Shreveport and Ochsner–Louisiana State University-Monroe facilities in Louisiana between March 17, 2020, and June 16, 2020. COVID-19 positivity was confirmed via nasopharyngeal swab and the standard polymerase chain reaction testing protocol established at the hospitals. Neither Ochsner–Louisiana State University-Shreveport nor Ochsner–Louisiana State University-Monroe has a dermatology service, so cutaneous manifestations were determined from clinical notes or descriptions in medical records (under the integument portion of the physical examination section) and from the use of *International Classification of Diseases* billing code R21, defined as rash and other nonspecific skin eruption.

## RESULTS

We identified 1,086 COVID-19–positive patients during the 3-month study period: 871 African Americans, 130 Caucasians, 41 Latinos, and 44 patients with unknown or other ethnicity. The cohort consisted of 529 females and 557 males, with a median age of 42 years.

Our review showed that 10 of 1,086 patients (0.9%) exhibited COVID-19–induced skin abnormalities: 6 of the 871 African American patients (0.7%) and 4 of the 130 Caucasian patients (3.1%). The incidence of cutaneous manifestations was 4.49 times higher in Caucasians than in African Americans. Demographic information about each patient and descriptions of their skin abnormalities are presented in the Table. The types of rashes noted were fine erythematous papules, burst blisters with superficial skin breakdown and epithelial sloughing, pruritic rash, diffuse vitiligo-like rash with fluid-filled blisters, erythematous peeling rash, and blanchable urticarial rash.

**Table.** Cutaneous Manifestations in COVID-19–Positive Patients, n=10

Race	Age	Sex	Rash Characterization	Location	Cause
African American	5 weeks	Male	Fine erythematous papules	Right side of face	Possible COVID-19 etiology
	49 years	Male	Burst blisters with superficial skin breakdown and epithelial sloughing	Scapula, posterior trunk, buttocks, posterior and left upper thigh	Frostbite from cooling blanket Possible COVID-19 etiology
	53 years	Female	Pruritic rash	Right upper extremity	Systemic lupus erythematosus (known history) Possible COVID-19 etiology
	64 years	Male	Diffuse vitiligo-like rash with fluid-filled blisters	Anterior and posterior trunk	Possible COVID-19 etiology
	68 years	Female	Skin breakdown, erythematous peeling rash	Neck and posterior trunk	Failure to thrive, negligence Possible COVID-19 etiology
	77 years	Female	Hypopigmentation	Anterior trunk	Possible COVID-19 etiology
Caucasian	29 years	Male	Erythematous macular rash with scaling	Bilateral upper extremities and palms, trunk, glabella	Syphilis detected Possible COVID-19 etiology
	43 years	Male	Burst blisters with skin sloughing	Anterior trunk	Possible COVID-19 etiology
	52 years	Female	Erythematous rash	Anterior trunk, bilateral upper extremity	Possible COVID-19 etiology
	57 years	Male	Blanchable urticarial rash	Anterior trunk, bilateral upper and lower extremity	Possible COVID-19 etiology

COVID-19, coronavirus disease 2019.

## DISCUSSION

The high rate of infectivity of severe acute respiratory syndrome coronavirus 2, the virus that causes COVID-19, resulted in a rapid spread across geographic boundaries, leading to a global pandemic.^8^ The clinical spectrum of COVID-19 ranges from nonapparent or mild symptoms to fatal forms with respiratory failure, septic shock, or multiorgan dysfunction.^9^ The common symptoms of patients with COVID-19 include cough, fever, sputum production, myalgias, fatigue, shortness of breath, sore throat, and headache.^10^ However, Henry et al suggest that COVID-19–induced cutaneous manifestations may serve as an indicator of infection before the incubation period of up to 14 days, aiding in timely diagnosis.^5^ Cutaneous manifestations have been reported in case reports and letters to the editor,^2-7,11-18^ but formal studies are lacking.

Recalcati reported a 20.4% incidence of cutaneous manifestations in 18 of 88 confirmed COVID-19–positive patients in Italy.^2^ Sachdeva et al conducted a literature review of 72 COVID-19–associated cutaneous manifestations and found that 12.5% of patients presented with cutaneous lesions before symptomatic onset of illness.^7^ Guan et al reported a 0.2% incidence of rash in 1,099 patients from China.^1^ The majority of our population was African American patients (80.2%), a racial prevalence that is not reflected in the literature about cutaneous manifestations of COVID-19.

Compared to African Americans, the incidence of skin abnormalities observed in our study was quadrupled in Caucasians. No scientific evidence supports the presence of intrinsic, individual-level factors or increased susceptibility to COVID-19 in various ethnicities.^19^ We postulate that the increased incidence of cutaneous manifestations in Caucasians vs African Americans is multifactorial. Social conditions such as limited health care access may result in the underdiagnosis of cutaneous manifestations in African Americans. Additionally, medical training is deficient with regard to identifying cutaneous manifestations in patients with dark complexions.^20,21^ We believe a larger number of cutaneous manifestations could have been identified if the medical literature increased awareness of rash presentations in different races and ethnicities. We challenge the medical literature to incorporate images of dermatologic processes in patients with dark complexions.

In our study, only 0.7% of the African American patients—1 infant and 5 adults—were documented to have skin manifestations. The infant exhibited fine erythematous papules on the right side of his face.

The mechanism for COVID-19–induced rashes in infants is not known; however, chilblain rashes seen in children with lupus erythematosus could be attributable to the antiviral and immunostimulatory properties of type I interferons (IFN-I).^18^ In adults, an inadequate or delayed IFN-I response can exacerbate preexisting hypercytokinemia, increasing morbidity and mortality.^18^

Viral infection with COVID-19 could present as lymphocytic vasculitis similar to the thrombophilic arteritis induced by immune complex–activated cytokines.^7,13^ The herpes virus is also known to activate interleukin-1, IFN-gamma, and tumor necrosis factor-alpha, leading to the recruitment of cytotoxic and natural killer cells that target the keratinocytes.^13^ The immune response to infection also leads to Langerhans cells activation, resulting in a state of vasodilation and spongiosis.^7,13^

Cutaneous manifestations can also be attributed to COVID-19–induced microthromboses originating in distal organs that may potentially attenuate blood flow to the cutaneous microvasculature and lead to manifestations resembling livedo reticularis.^22,23^ Low-grade disseminated intravascular coagulation and hypoxia-related accumulation of deoxygenated blood in venous plexuses may further explain such manifestations.^1,2,23^ Magro et al observed extensive complement involvement, membrane attack complex–mediated microvascular endothelial cell injury, and subsequent activation of a clotting pathway, leading to fibrin deposition in patients with severe COVID-19 pneumonitis.^24^ Further, COVID-19 viral infection could have a vasculitis component. Nevertheless, whether cutaneous symptoms are a secondary consequence of respiratory-related infection or a primary infection of the skin itself remains unclear. A combination of such mechanisms is likely responsible for the cutaneous manifestations in COVID-19–positive individuals.^7^

This study has limitations. Cutaneous manifestations are often more difficult to appreciate in African Americans than Caucasians because of the lack of contrast between the rash and the skin color. The lack of a dermatology service at our institutions could have limited our findings. The information for this study was obtained from medical record review. Patients may not always report a rash, and hospital staff may forget to ask about cutaneous involvement. Because of the high virulence of COVID-19, full-body examinations of the COVID-19–positive patients could have been less thorough compared to hospitalized patients without COVID-19. The cutaneous manifestations identified in this study could have been attributed to medications, allergies, and long-standing dermatologic cutaneous involvement. Patients included in this study were admitted during a peak in the COVID-19 pandemic in the United States, explaining the short study period of 3 months.

## CONCLUSION

Our data provide evidence that cutaneous manifestations of COVID-19, especially in African American patients, are rare, but documenting such cases is necessary to establish a cause and effect for COVID-19–induced skin manifestations.

## Notes

*This article meets the Accreditation Council for Graduate Medical Education and the American Board of Medical Specialties Maintenance of Certification competencies for Patient Care and Medical Knowledge.*
